# Sanda training for youth athletes: a comprehensive review of health benefits, injury prevention, and long-term development

**DOI:** 10.3389/fspor.2026.1797232

**Published:** 2026-05-07

**Authors:** Yong Jiang, Wenyang Su, Tiezhu Niu, Lei Ma

**Affiliations:** 1 School of Kinesiology and Health Promotion, Dalian University of Technology, Dalian, China; 2 School of General Education, Dalian University of Technology, Panjin, China; 3 Gymnasium of Physical Education Teaching Department, Dalian Minzu University, Dalian, China

**Keywords:** athlete development, injury prevention, martial arts training, physical health benefits, Sanda, youth athletes

## Abstract

**Background:**

Sanda is a dynamic and practical Chinese martial art that was developed as a modern combat sport in the late twentieth century by the Chinese People's Liberation Army, drawing on traditional kung fu and contemporary close-combat methods. Sanda emphasizes a combination of full-contact punching, kicking, throwing, and clinch-control techniques, making it distinct from many other striking-based martial arts.

**Objectives:**

This review examines several benefits of Sanda training for youth athletes, including improvements in physical health, intellectual resilience, and talent.

**Methods:**

We searched PubMed, Web of Science, Scopus, and CNKI from 2015 to October 2025, identifying records; after screening and applying inclusion criteria, studies on youth Sanda or closely related combat sports were retained for narrative synthesis.

**Results:**

The article discusses the most common accidents related to Sanda, providing strategies to prevent damage and the role of recovery. It also highlights the importance of vitamins and rest for optimal overall performance and long-term athletic development. Ultimately, we examine Sanda's effectiveness compared with other martial arts and suggest areas for future research to enhance educational methods, safety protocols, and overall performance tracking.

**Conclusions:**

Through an integrative approach to education and development, Sanda offers youth athletes a stable foundation for success in martial arts and the past.

## 1 Introduction

Sanda, additionally called Sanshou (1), is a dynamic and multifaceted Chinese fight sport that integrates striking, kicking, wrestling, and throwing strategies (2). Originating as a military hand-to-hand combat education device inside the People's Liberation Army (PLA), it advanced through the incorporation of traditional Chinese martial arts and modern-day fight systems, such as kickboxing and wrestling (3). Its large appeal lies in its mixture of both offensive and protective techniques, making it a versatile schooling technique that draws athletes from various martial arts backgrounds (1).

In terms of psychological blessings, Sanda performs an essential function in cultivating intellectual resilience, recognition, and emotional balance. Youth athletes engaged in Sanda schooling are continuously confronted with situations that test their capacity to perform under stress. Whether in sparring periods, opposition, or excessive education drills, Sanda teaches athletes to alter their feelings, stay focused, and build the mental fortitude needed to be successful in sports and life (4, 5). Studies (6) suggest that martial arts like Sanda offer significant cognitive and psychological benefits, particularly in terms of concentration and stress management.

But, like all contact sports, Sanda does bring inherent injury risks, specifically for youth athletes. The sport's emphasis on high-depth putting, dynamic kicks, and aggressive takedowns increases the likelihood of muscle strains, sprains, and joint injuries (7). Commonplace injuries consist of those to the knee, ankle, shoulder, and neck, especially because of the bodily demands of throwing and hanging (8). These risks underscore the significance of injury prevention strategies, including proper methods, warm-ups, cool-downs, and the use of appropriate shielding gear. Furthermore, training depth needs to be customized according to the athlete's age and developmental stage to minimize the risk of overuse injuries and ensure long-term fitness.

Nutrition and recovery are also critical components for youth athletes' education in Sanda. The dietary needs of youth athletes in high-depth sports, which encompass Sanda, require cautious planning to ensure they gather sufficient energy, macronutrients, and micronutrients (9). The right vitamins help usual performance, muscle healing, and widespread growth. Carbohydrates provide the energy vital for intense training, at the same time as proteins are crucial for muscle repair (10). Furthermore, adequate hydration and the replenishment of electrolytes are vital in maintaining energy levels and lowering fatigue (11). Recovery is equally important in preventing overtraining and lowering the risk of damage. Relaxation days, energetic recuperation, and sleep all contribute to muscle repair and mental rejuvenation. The mixture of powerful dietary strategies and proper healing techniques guarantees that youth athletes can perform at their pleasant while minimizing the risk of burnout or damage (12).

As the popularity of Sanda continues to grow, especially among youth athletes, it's vital to comprehend its complete range of benefits and the challenges it poses. Key regions that require similar interest in future studies encompass the improvement of advanced education protocols, protection measures, and overall performance tracking in kids' Sanda packages. Moreover, exploring the function of go-education between Sanda and other martial arts ought to enhance athletes' universal fight talent, damage prevention, and mental conditioning (13). A deeper know-how of these elements will make sure that Sanda stays a secure, powerful, and holistic method to children's athletic development within the martial arts area (14).

This narrative review will summarize what is known about the physical, mechanical, and performance characteristics of Sanda athletes, examine how training and weight control influence these attributes, and provide some suggestions and output for future research to promote improved health and performance in the sport of Sanda.

## 2 Methods

This narrative review identifies and synthesizes peer-reviewed research relevant to Sanda and all closely associated Chinese combat sports. The level of aerobic and anaerobic power required to compete successfully in these combat sports is high, requiring professional weightlifters/instructors, competitive martial artists, professional soccer players, and others who possess both physical and psychological strengths, as well as speed and endurance. A systematic electronic search was performed between 2015 and October 2025 using PubMed, Web of Science, Scopus, CNKI, and combinations of keywords including “Sanda,” “Sanshou,” “Chinese kickboxing,” “combat sports,” “physiology,” “biomechanics,” “performance,” “training,” “injury,” and “weight control” to identify research papers and other peer-reviewed articles published during that time. Eligible studies were defined as those: (1) containing competitive Sanda athletes, (2) containing at least one of the following outcomes: physiological, biomechanical, performance, injury, and/or weight control, and (3) published as full-text papers in either English or Chinese. Studies included experimental, cross-sectional, and descriptive types to ensure that a comprehensive overview of the literature was included. The sample characteristics, the level of competition, methodology and key findings from each included study were extracted for thematic analysis, including: (a) the physical and physiological characteristics of competitive Sanda athletes; (b) the biomechanical and tactical characteristics of the sport; (c) the types of training and conditioning interventions used by competitive Sanda athletes; and (d) the types of injury prevention and weight control methods used by competitive Sanda athletes.

Through the systematic search of PubMed, Web of Science, Scopus, and CNKI (2015–October 2025), we screened titles, abstracts, and full texts of studies on competitive Sanda and related Chinese combat sports. Studies that met all inclusion criteria and reported relevant physical, physiological, injury, or weight-management outcomes in competitive Sanda athletes were included in the final narrative synthesis.

## 3 Application in modern sports and training

Its integration of hanging, kicking, and throwing strategies makes it a versatile recreation, attracting athletes from numerous martial arts backgrounds (15). Within the MMA community, fighters with a historical past in Sanda often excel due to their skill ability in stand-up striking and managing in the clinch, regions which can be crucial in combined combat environments (16).

Athletes undergo rigorous schooling to develop their energy, velocity, and persistence. Education includes sparring, in which practitioners simulate real opposition situations with an emphasis on keeping speed and agility throughout the fight. Moreover, Sanda education makes a specialty of intellectual longevity, coaching combatants to remain focused and disciplined in the course of excessive-stress situations (17).

Furthermore, sports activities and medicinal drugs play a critical role in harm prevention and rehabilitation, given the sport's bodily traumatic nature. Sanda's emphasis on sensible fighting techniques additionally makes it a super choice for self-protection training. Its versatility allows practitioners to build foundational talents that may be applied in real-world eventualities. Self-defense schools and martial arts academies across the globe have followed Sanda elements to teach students powerful strategies for managing confrontations, from avenue fights to more established altercations (18).

## 4 Impact of sanda on physical health

Sanda has received a reputation as an effective combat sport that gives numerous physical health benefits to youth athletes. As summarized in Table 1, multiple studies consistently show that Sanda training in youth improves muscular strength, cardiovascular fitness, flexibility, and body composition, although these benefits must be balanced against injury risk. Studies have shown that ordinary participation in Sanda education undoubtedly affects numerous aspects of physical health, including muscular strength, cardiovascular health, flexibility, and staying power (1, 16).

**Table 1:** Sanda's impact on physical health for youth athletes.

Physical health aspect	Impact of Sanda	Benefit to youth athletes	Disadvantages	References
Muscular strength and endurance	Sanda improves muscular endurance and strength through dynamic movements like striking, kicking, and throwing	Enhances muscle tone, stability, and overall strength	Risk of overtraining, leading to muscle fatigue or injury	(19, 20)
Cardiovascular health	Intense training in Sanda enhances cardiovascular fitness, improving heart health and oxygen utilization during exertion	Boosts stamina and heart health, improving overall fitness	High-intensity training can be demanding for youth athletes	(21, 22)
Flexibility and coordination	Sanda incorporates kicks and footwork that improve flexibility and develop balance and coordination	Promotes motor development and athleticism in youth athletes	Overstraining in flexibility can lead to joint injuries	(23, 24)
Injury prevention	Sanda's emphasis on correct technique and dynamic movement helps prevent injuries, particularly in the knee, ankle, and shoulders	Reduces injury risks and promotes safe training and movement	Contact injuries during sparring, despite safety protocols	(25, 26)
Body composition and weight management	Sanda training increases muscle mass while decreasing body fat, contributing to a healthier body composition	Encourages healthy weight management and enhances physical appearance	Intense training without proper nutrition may lead to fatigue	(27)

### 4.1 Muscular strength and endurance

The physical conditioning of martial artists discovered that Sanda athletes display full-size improvement in muscular strength and persistence as compared to athletes from non-combat sports activities. This is attributed to the high-depth nature of the sport, which involves rapid, powerful moves, including punches, kicks, and throws (28). These actions set off a couple of muscle groups, building each upper and lower body power. For youth athletes, this no longer most effectively enhances muscle tone but additionally improves basic bodily performance (29).

In practical terms, repeated combinations of punches, roundhouse kicks, and explosive hip-driven throws require coordinated activation of the lower–limb musculature, trunk stabilizers, and upper–body prime movers, progressively increasing whole–body strength and muscular endurance in youth athletes. Typical youth Sanda sessions involve multiple rounds of pad work, partner drills, and controlled sparring, which together generate a substantial cumulative loading stimulus; however, if weekly training volume or intensity is increased too rapidly, overuse problems such as shoulder impingement, lumbar fatigue, and knee pain may emerge.

### 4.2 Cardiovascular health

Cardiovascular advantages found in youth practitioners of combat sports activities like Sanda (30). The findings highlighted that Sanda schooling drastically improves cardiovascular performance, leading to better coronary heart health and oxygen usage at some point of extreme bodily exertion. The aerobic ability of Sanda athletes is greater because of the game's emphasis on extended, high-depth exertion periods at some stage in sparring and exercise. As a result, youth athletes engaged in Sanda can revel in higher cardiovascular fitness and stamina, which is crucial for normal health (31).

From a cardiovascular perspective, standard Sanda rounds (2–3 min bouts interspersed with short rest intervals) closely resemble high-intensity interval training, with repeated spikes in heart rate into vigorous or near-maximal zones followed by partial recovery. Such work–rest structures stimulate both aerobic and anaerobic energy systems, improving maximal oxygen uptake, cardiac output, and the ability to tolerate high-intensity efforts in youth athletes. At the same time, if conditioning loads are not age-appropriate such as excessive numbers of continuous hard rounds without adequate rest, youth athletes may experience undue fatigue, autonomic imbalance, or reduced motivation, underscoring the need for careful monitoring of perceived exertion and recovery.

### 4.3 Balance, coordination, and flexibility

One of the foremost physical benefits of Sanda schooling is the development of stability and coordination, which is due to the dynamic movements required in the sport, including complicated footwork, spinning kicks, and short directional adjustments. Research has shown that youth athletes who exercise Sanda increase advanced motor management, which translates to higher balance and coordination both inside and outside of the sports environment (32). Moreover, the game's emphasis on flexibility, sporting events to execute high kicks, and numerous stretches helps youth athletes improve their normal variety of motion, which could help reduce injury risk and enhance physical performance. Complex technical skills such as spinning back kicks, jumping roundhouse kicks, and catching and sweeping an opponent's lead leg challenge dynamic balance, proprioception, and rapid postural adjustments, thereby refining neuromuscular control in growing athletes. Repeated practice of these movements, combined with controlled break-falls and direction changes, helps youths develop more efficient movement strategies that transfer to everyday tasks and other sports requiring agility and coordination. However, if these advanced drills are introduced too early or performed with poor supervision, the high rotational and impact forces at the hip, knee, and ankle may increase the risk of joint irritation or soft-tissue strain.

### 4.4 Body composition and weight management

Studies have also observed that Sanda education can positively impact body composition. According to researchers, adolescent Sanda practitioners confirmed a better percentage of lean muscular tissues, and a lower body fat percentage as compared to non-athletes (33). This is partly because of the excessive calorie burn and excessive physical activity required during the Sanda schooling periods. The sport encourages weight management through rigorous physical exertion, making it useful for selling a wholesome way of life among kids athletes (34). Frequent high-intensity Sanda sessions substantially increase daily energy expenditure and create repeated periods of elevated metabolic demand, which, when combined with adequate nutrition, can reduce body fat and promote favorable gains in lean mass during adolescence. These adaptations support healthier body composition profiles and may counteract sedentary lifestyle tendencies common in youth populations. Nevertheless, if youth athletes imitate adult combat-sport practices such as rapid weight cutting, severe energy restriction, or dehydration before competition, they may compromise growth, hormonal balance, and training quality, highlighting the importance of coach and parent education on age-appropriate weight-management strategies.

### 4.5 Real-life athletic development through sanda

The blessings of Sanda for youth athletic improvement are evident in numerous actual-life instances of athletes excelling in their sports, especially in mixed martial arts (MMA), kickboxing, and Wushu competitions. The schooling methodologies and bodily conditioning supplied by Sanda have proven to be instrumental in shaping successful athletes in those disciplines (35).

**Figure 1:**
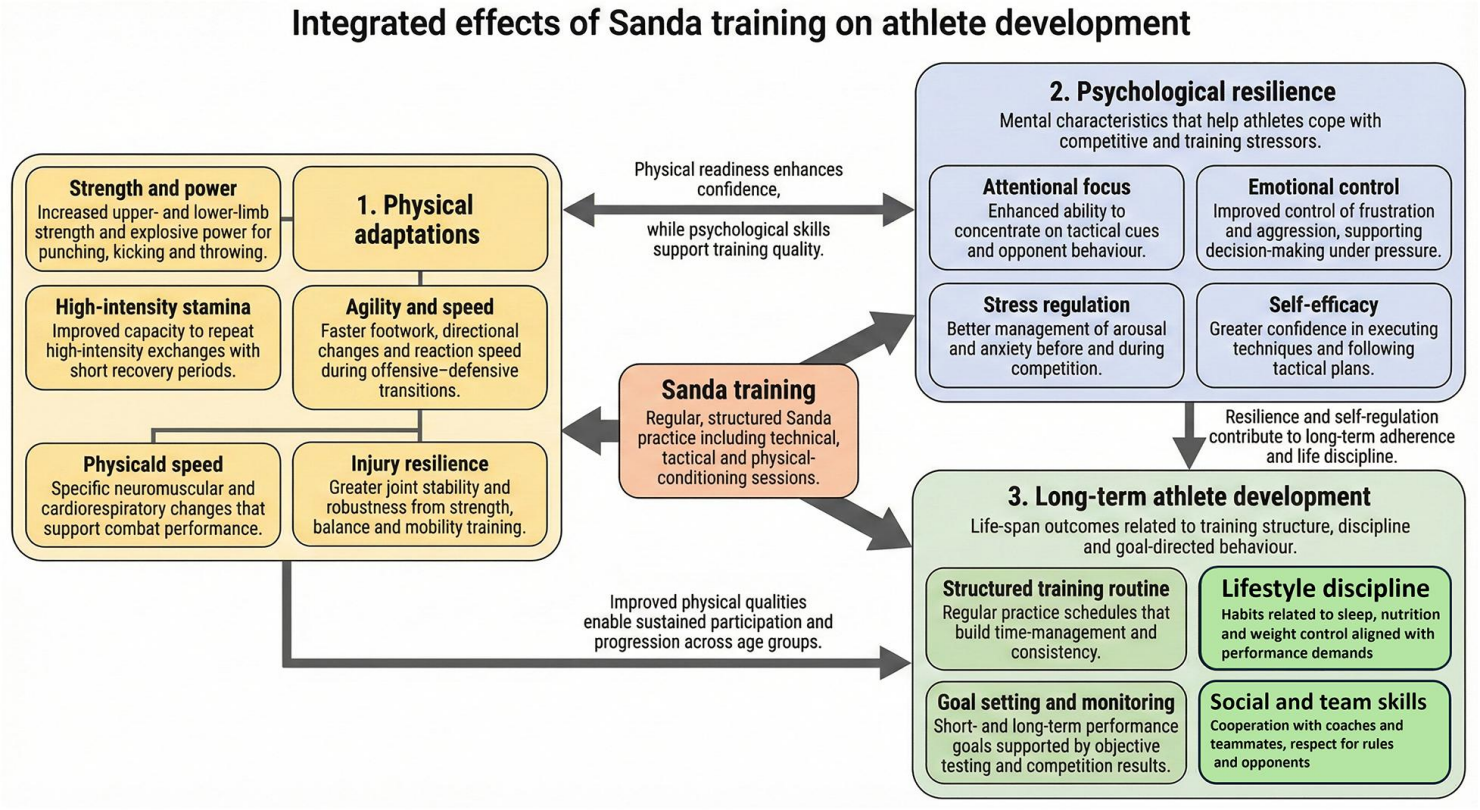
Key benefits of Sanda training for youth athletes include improvements in physical well-being, psychological resilience, and overall developmental progression.

In China, many adolescent sports academies include Sanda schooling in their martial arts applications. These programs intend to foster bodily improvement and mental toughness among youth athletes. The Chinese language countrywide Wushu crew, which trains youth athletes in numerous disciplines, including Sanda, has produced several champions who excel in both countrywide and worldwide competitions. The training focuses not only on technical mastery but also on building resilience, which is essential for success in high-strain athletic environments (36). Sanda has been beneficial in beginner sports clubs and adolescent schooling programs. A case study of a newbie Sanda education group in Shanghai discovered that after one year of constant education, child participants verified advanced bodily fitness markers, including elevated aerobic capability, higher muscular endurance, and a significant reduction in body fat percentage. Those findings recommend that Sanda may be an effective game for enhancing the overall bodily fitness of youth athletes, even in non-aggressive settings (37). Figure 1 illustrates the primary benefits of Sanda training for youth athletes. The first category, Physical well-being, highlights key improvements such as strength, stamina, and agility. The second category, Psychological Resilience, demonstrates the impact of Sanda on athletes' mental health, including better focus, stress regulation, and emotional control. Lastly, Developmental Progression emphasizes the broader life skills fostered by Sanda, such as structured training, goal orientation, and discipline, all of which are critical for personal growth and success both in and outside the sports environment.

## 5 Psychological and cognitive benefits

One of the primary psychological benefits of Sanda is the development of mental resilience. In fight sports activities, athletes are often exposed to worrying conditions, whether through sparring, competition, or intense training. The ability to hold composure under pressure is critical, and Sanda schooling encourages resilience through regular intellectually demanding situations (38). It is found that combat athletes, mainly those in Sanda, confirmed a higher degree of emotional durability and flexibility as compared to athletes in non-fight sports activities. This resilience helps them cope not only with the physical needs of schooling but also with challenges outside the sport, contributing to their overall mental well-being (39).

Sanda education requires athletes to maintain constant focus throughout both defensive and offensive moves. Recognition is especially emphasized in strategies such as striking, kicking, and throwing, in which brief reactions and particular movements are vital for fulfillment. Sanda athletes showed sizable upgrades in their awareness and reaction instances at some stage in high-pressure situations. This capability to preserve consciousness under duress is a cognitive ability that extends beyond the training surroundings, benefiting youth athletes in college, work, and other aspects of life (40). Moreover, intellectual subjects learned from Sanda training enable athletes to remain active in the second and keep away from distractions, which is essential for each performance and fashionable intellectual fitness. Emotional law is another key benefit associated with Sanda. Combat sports activities like Sanda require athletes to stabilize aggression with manipulation, preserving perfect emotional control throughout the competition (41). The high-depth nature of Sanda often evokes strong feelings, including anger, frustration, or tension; however, athletes are trained to control those emotions and cognize them into effective fight techniques. The athletes training in Sanda displayed greater emotional stability compared to non-athletes. The rigorous training, blended with the established nature of Sanda competitions, facilitates youth athletes to discover ways to cope with both victory and defeat positively, leading to advanced emotional intelligence (42).

### 5.1 Connection between physical activity and mental health in martial arts

The connection between physical activity and mental fitness is well documented within the literature, and martial arts like Sanda provide a unique platform for improving both. Studies consistently show that conducting physical activity, based on sports like martial arts, has a profound impact on mental health, decreasing signs of stress, anxiety, and depression at the same time as boosting overall well-being (43).

Within this context, the structured, contact-based training environment of Sanda exposes youths to repeated, manageable stressors such as facing an opponent, coping with physical contact, and performing under observation, which can gradually strengthen coping skills and perceived self-efficacy. Regular participation in such sessions encourages athletes to use functional strategies (breathing control, attentional focus, and problem-solving) to manage arousal, aligning with broader evidence that appropriately dosed physical activity supports reductions in stress, anxiety, and depressive symptoms in children and adolescents. However, if training intensity, competition frequency, or coach expectations exceed a youth athlete's developmental capacity, these same stressors may become overwhelming, emphasizing the need for supportive coaching practices and open communication to maintain a positive mental-health trajectory.

### 5.2 Stress reduction and anxiety management

Physical workout, such as martial arts, has been shown to lessen strain by releasing the release of endorphins. Adolescent contributors in martial arts, along with Sanda, exhibited a sizeable reduction in tension levels after normal training classes. That is mainly essential for youth athletes, as they often face educational and social pressures, which can result in pressure and anxiety. The subject and consciousness required during Sanda education help youth athletes manage these pressures, improving their mental resilience and equipping them with higher pressure-coping strategies (44). In Sanda, repeated exposure to sparring, controlled contact, and competitive simulations provides a natural laboratory for practising emotion regulation, as youths must manage fear, frustration, and excitement while still executing techniques effectively. Over time, successfully navigating these situations can reduce performance-related anxiety and help athletes generalize calm-under-pressure skills to school, social contexts, and other sports. Nonetheless, if coaches rely heavily on punishment, humiliation, or constant comparison between athletes, training may increase anxiety rather than alleviate it, so psychologically informed coaching that emphasizes mastery, effort, and safety is critical to realize the stress-reducing potential of Sanda.

### 5.3 Depression prevention and improved mental well-being

Martial arts have also been proven to have depressive symptom-reducing consequences. Youth athletes who practiced martial arts, which include Sanda, had a lower level of depression as compared to their peers who did not engage in daily physical activity. The mixture of physical exertion, intellectual awareness, and social interplay at some stage in training contributes to a sense of well-being. The social aid found inside Sanda training agencies, in which athletes interact with coaches and fellow athletes, additionally gives emotional advantages, lowering emotions of loneliness or isolation (45).

Taking part in Sanda enables building an experience of achievement through physical mastery and competition. The capability to carry out hard strategies, win fits, and acquire feedback from coaches can notably raise youth athletes. Athletes in martial arts, which includes Sanda, exhibited better degrees of self-belief and self-efficacy. Those traits no longer best enhance athletic performance but also translate into better academic results and advanced social relationships, as youth athletes sense more successful and empowered in other factors in their lives (46). Beyond acute mood benefits, the long-term process of learning Sanda techniques, earning recognition from coaches, and progressing through competitive experiences can foster a stronger sense of competence and social connectedness, both of which are protective factors against depressive symptoms in youths. The supportive team environment often found in Sanda clubs, where athletes train together, share challenges, and celebrate improvements may further reduce feelings of isolation and enhance overall life satisfaction. However, when emphasis is placed exclusively on winning or on maintaining a certain body image, vulnerable youth athletes may experience increased negative self-evaluation, so balancing performance goals with holistic well-being should be a central principle in youth Sanda programs.

## 6 Injury risks and safety measures

The character of Sanda, which combines striking, kicking, throwing, and grappling, exposes athletes to diverse kinds of injuries, especially those related to impact and physical exertion. The most common injuries in Sanda contain contusions, sprains, and lacerations because of striking and kicking. Knee accidents are especially common due to the excessive frequency of kicking and knee movements, often leading to ligament damage or patellar tendinitis. Moreover, ankle sprains are common because of the excessive-velocity sidekicks and spinning kicks, which require specific foot placement and might lead to twisting or overextension (47).

Head injuries, mainly concussions and facial injuries, are a subject in any striking recreation, and Sanda is no exception. Using punches and kicks to the head and face makes it critical for athletes to wear defensive gear, along with headguards and mouthguards, at some stage in training and competition. Neck accidents, along with lines and whiplash, can occur from throwing strategies, especially while an athlete is taken to the ground during a takedown or throw (48). Repetitive movements, particularly in youth athletes who are nevertheless growing physically, can cause overuse injuries. Overuse injuries, along with tendinitis or pressure fractures, are commonplace in fight sports activities like Sanda, specifically while athletes over-train without adequate relaxation periods. Shoulder accidents and elbow tendonitis are commonplace in athletes who regularly use punching and grappling techniques without suitable recovery (49).

## 7 Importance of warm-ups, cool-downs, and injury management techniques

### 7.1 Warm-ups

The importance of warm united states cannot be overstated in preventing accidents in Sanda. A proper heat-up increases blood flow to muscle mass, improves joint mobility, and prepares the athlete mentally for training. Dynamic stretching, inclusive of leg swings, arm circles, and hip rotations, enables the activation of the muscular tissues and prepares them for high-intensity actions like kicks, punches, and throws. Additionally, light aerobic sporting events, which include running or skipping, can increase coronary heart flow and frame temperature, which complements performance and decreases the chance of muscle strains and ligament sprains at some point in education (50).

### 7.2 Cool-downs

**Figure 2:**
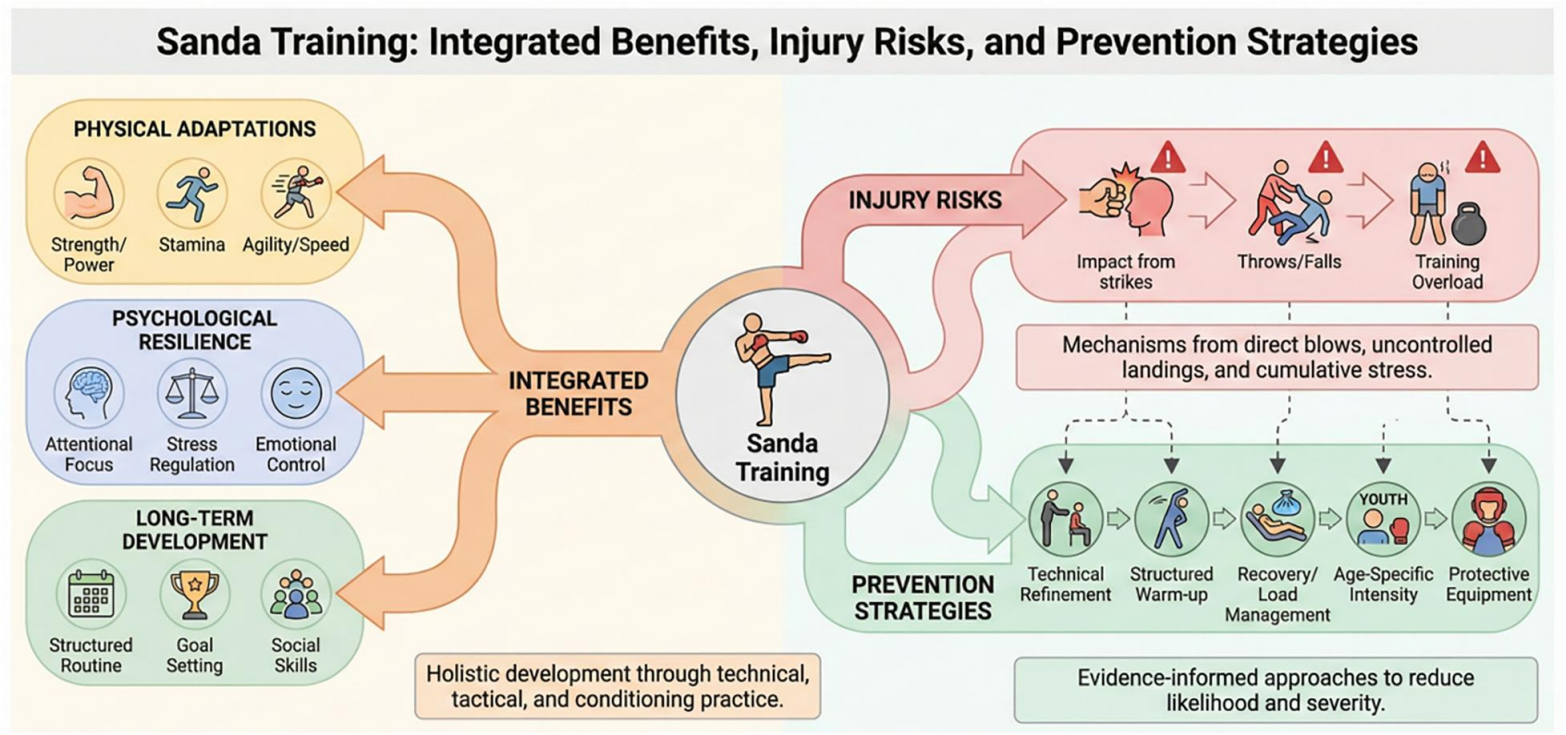
Common injury risks associated with Sanda training and recommended injury prevention methods.

Just as warm-ups are crucial, cool-downs play a key role in injury prevention and recovery. After a strenuous Sanda session, a cool-down consisting of static stretching helps to maintain flexibility and prevent muscles from becoming stiff or tight. Static stretches for the hamstrings, quadriceps, and calves can help release muscle tension built up during the workout (51). Furthermore, cooling down allows the body's heart rate to gradually return to normal and can promote lactic acid removal, reducing the likelihood of delayed muscle soreness. Figure 2 highlights the primary injury risks in Sanda training, including strikes and kicks, throws and takedowns, and the potential for overtraining and repetitive movements. It also provides key injury prevention methods, such as proper warm-ups and mobility drills, cool-down stretches, and adjusting training intensity based on age to minimize injury risks.

### 7.3 Injury management techniques

In the event of damage, proper first aid and rehabilitation protocols are crucial to ensure complete recuperation and avoid lengthy-time period damage. The R.I.C.E. (rest, Ice, Compression, Elevation) method is widely endorsed for coping with acute injuries such as sprains and strains. Immediate ice application allows for the reduction of swelling and infection, while compression and elevation can further alleviate these symptoms. More serious accidents, which include fractures or ligament tears, require clinical assessment and feasible imaging to determine the extent of the harm (52). For long-term injury prevention and rehabilitation, athletes can benefit from physical therapy, which specializes in strengthening susceptible regions, improving joint mobility, and providing targeted sports to address unique injury-inclined zones (including the knees, ankles, and shoulders) (53). Moreover, retaining a balanced schooling routine that consists of good enough relaxation days and pass-schooling can prevent overuse injuries and help athletes avoid burnout.

## 8 Training practices for youth

**Figure 3:**
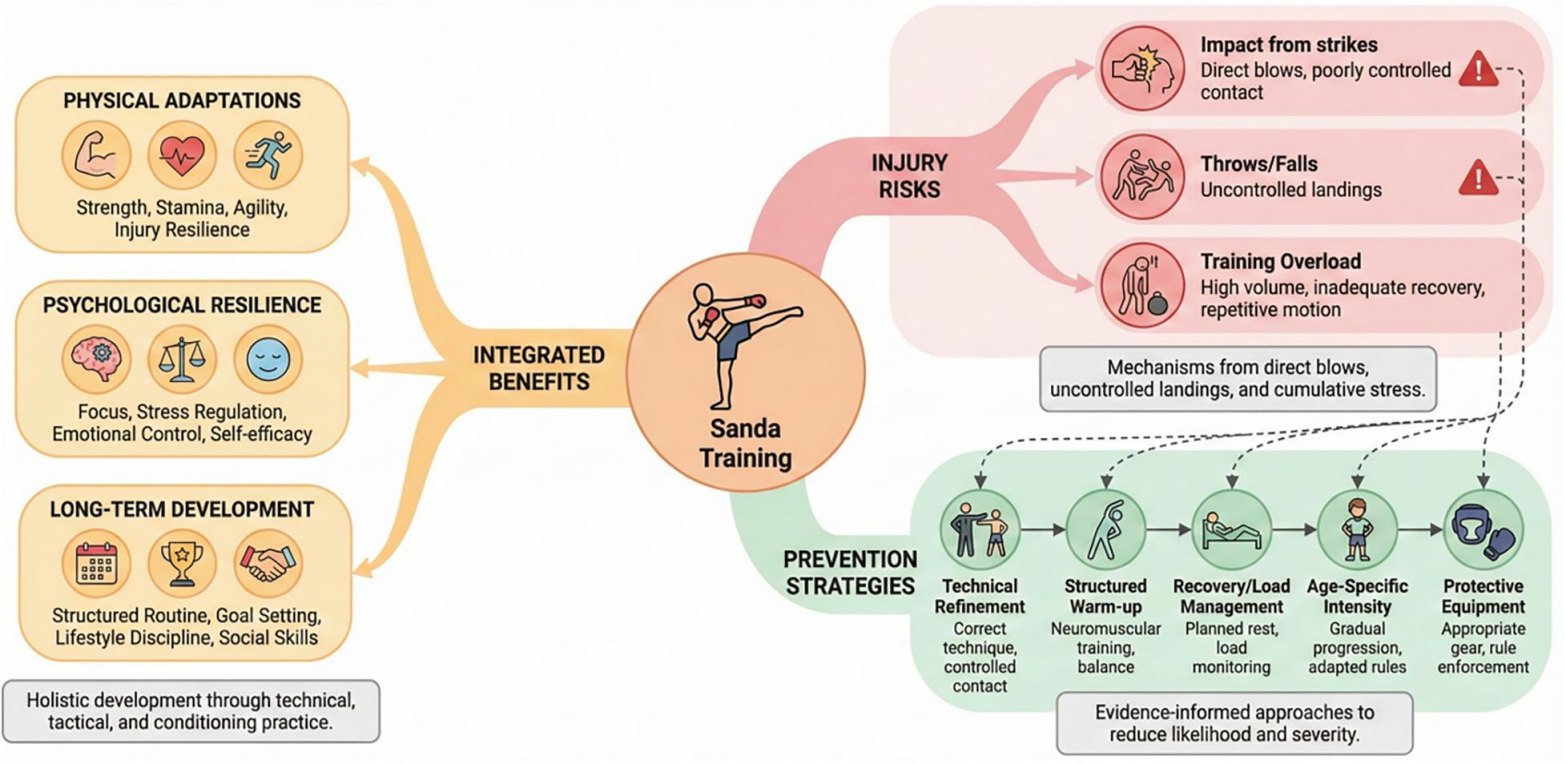
Age-appropriate training strategies for Sanda athletes across different age groups.

For youth athletes, Sanda training must be designed to deal with their developmental needs, focusing not only on the technical elements of the game but also on promoting physical health, intellectual resilience, and long-term athletic improvement. A perfect Sanda training agenda for youth athletes generally carries a mixture of electricity and conditioning, technical drills, and relaxation periods, aiming to construct a sturdy foundation while minimizing the risk of overtraining and harm (54). Figure 3 outlines age-appropriate training strategies for Sanda athletes. For youth athletes (6–12 years), the focus is on skill basics and play-based activities. For mid-teens (13–15 years), the training incorporates more technique drills and emphasizes physical fitness. In the older age group (6–18 years), training shifts to sparring, conditioning, and preparing athletes for competitive readiness.

### 8.1 Frequency of training

The frequency of training for children athletes varies primarily based on their age, physical development, and athletic desires. For youth athletes (ages 6–12), 3–4 education sessions in step with the week are typically enough to allow for each talent's improvement and bodily conditioning. Those periods ought to be a mixture of laughter and interactive drills with an emphasis on gaining knowledge of techniques and building simple energy. For older youth athletes (aged 13–18), who can be competing at a better level, 4–6 training classes per week can be necessary to enhance patience, power, and competition readiness. Those education schedules need to step-by-step increase the depth as the athlete matures. The key is to keep away from overloading the body, as this could cause accidents or burnout (55).

### 8.2 Duration of training

The training periods for youth athletes ought to normally range from 60 to 90 min, depending on the athlete's age and development stage. For youth, shorter, extra-targeted periods (45–60 min) are advocated to hold their attention and energy levels high. These periods must integrate technical drills, games, and bodily conditioning physical activities. As athletes develop and mature, the session period can grow, with greater time devoted to precise aspects of the game, which include sparring, throwing techniques, and conditioning sporting activities. For older athletes, 90 min to two-hour periods may be appropriate, considering more in-depth schooling and more time for talent refinement (54).

### 8.3 Recovery and test

Rest and recuperation are important components of an education schedule, mainly for youth athletes. The depth of Sanda education can be physically disturbing, so it's vital to balance difficult schooling with adequate restoration time. Youth athletes need to have at least one full relaxation day consistent with the week to permit the body to recover and adapt to the schooling stimulus. Incorporating active restoration, including mild stretching or swimming, can also be beneficial for muscle restoration without compromising overall performance (56).

### 8.4 Customization of training intensity based on age and physical development

As the youth athletes progress in their Sanda schooling, it's essential to personalize their education intensity consistent with their age, physical improvement, and character development. Overloading youth athletes with excessive intensity or extent too early can increase the danger of damage and preclude long-term development. Here's how education depth can be adjusted: At this age, the focus should be on essential motor abilities, basic methods, and developing coordination and flexibility. Schooling depth should be kept exceedingly low, with a focus on a laugh and varied physical games that introduce youth athletes to special factors of fight sports. The extent of schooling needs to be slight, with an emphasis on skill improvement in place of intensity. Sessions need to encompass balance drills, footwork, and primary putting strategies to help set up a strong basis for future athletic overall performance (57).

For youth in this age group, training intensity can gradually increase. The emphasis ought to be on building energy, enhancing persistence, and refining strategies in all elements of Sanda, which include hanging, kicking, and throwing. While youth athletes are still developing bodily, it is vital to raise awareness on proper methods and motion patterns to avoid injuries. At this degree, strength education can be introduced in a controlled way, which specializes in body weight physical activities and occasional-resistance electricity education (58).

## 9 Role of nutrition and recovery in sanda training for youth athletes

The right nutrients play a critical role in the overall performance, recovery, and universal fitness of youth athletes' education in Sanda. Given the intense physical needs of putting, kicking, and throwing, in addition to the need for explosive energy at some stage in training and competition, a balanced weight loss program is important for fueling the body and promoting optimal recovery (59).

### 9.1 Macronutrients

For youth Sanda athletes, carbohydrates are the number one source of energy, mainly during high-intensity training classes. Complete grains, fruits, vegetables, and legumes ought to be emphasized as the number one sources of carbohydrates, offering sustained strength for patience and intensity. Consistent with an examination, carbohydrate must account for 45%–65% of an athlete's daily caloric intake to support severe education and competition demands (60). Proteins are vital for muscle repair and restoration. For youth athletes, the advocated protein consumption must be around 1.2–2.0 grams per kilogram of body weight, depending on the depth of training. Resources of lean protein, which include chicken, fish, tofu, and legumes, must be integrated into food to support muscle restoration and increase. Fats, especially healthy fats from sources like avocados, nuts, and olive oil, assist long-term energy needs and are essential for the absorption of fat-soluble vitamins. Fats need to make up 20%–35% of the overall caloric consumption to support both overall performance and standard fitness (61).

### 9.2 Micronutrients and hydration

Essential nutrients and minerals, especially iron, calcium, and vitamin D, are crucial for bone health and energy production. Youth Sanda athletes have to make a certain good enough intake of iron (from sources like spinach, red meat, and lentils) to aid oxygen delivery and power metabolism through intense exercise. Calcium and vitamin D are essential for preserving bone density, which is specifically vital during periods of growth (62). Hydration is another key element in preserving performance and recovery. Youth athletes should aim to drink water during the day, with a focus on electrolyte replenishment post-training, and the use of liquids that include sodium and potassium to replace lost minerals through sweat. The advocated fluid intake is around 1.5–2 L consistent with day, with an extra 500–750 mL consumed at some stage in or after in-depth training classes.

## 10 Importance of recovery and rest in training programs

Restoration and relaxation are as vital to a youth athlete's development as the training itself. Extreme physical activity, in a high-contact game like Sanda, places strain on the muscle groups, joints, and nervous system, making it vital for athletes to combine appropriate restoration practices into their education timetable. The physical demands of Sanda, with its emphasis on aerobic and anaerobic physical games, require muscle healing to prevent overtraining and injuries. The R.I.C.E. method is generally used for dealing with acute injuries like strains and sprains. For muscle recuperation, rest days and active recuperation methods consisting of mild strolling, swimming, or yoga are beneficial for lowering muscle anxiety and promoting circulation without inflicting fatigue (63).

Sleep is a regularly omitted, however critical component of restoration. Sleep plays an important role in muscle recovery and the replenishment of glycogen stores, which might be depleted after excessive training. Youth athletes must aim for a minimum of 8–10 h of sleep per night, specifically throughout heavy education cycles. Proper sleep not only aids in physical restoration but also allows for intellectual cognizance and emotional control, which are crucial for overall performance in combat sports activities (64). Stretching is crucial for maintaining flexibility and preventing damage, especially in a game like Sanda, wherein flexibility is necessary for excessive kicks and explosive moves. A foam rolling duration that includes static stretching of the hamstrings, quadriceps, and calves is critical to reduce muscle tightness and improve flexibility. Moreover, incorporating foam rolling or massage therapy can help release muscle knots and enhance blood flow to the muscle groups, promoting quicker recovery (65).

For youth Sanda athletes, long-term improvement ought to focus on regularly growing training intensity even as emphasizing proper approach, physical conditioning, and mental durability. An extended-term athlete development (LTAD) model gives an established pathway for growth, ensuring that youth athletes progress at a safe and sustainable tempo. Early specialization in a single game like Sanda can lead to physical burnout and mental fatigue. Professionals suggest a multi-recreation technique throughout the early years of schooling, allowing youth athletes to increase an extensive range of movement skills and athleticism. By way of taking part in other sports, inclusive of swimming, track and field, or crew sports, youth athletes can construct a well-rounded athletic foundation, with a view to enhancing their long-term overall performance in Sanda.

## 11 Discussion

This narrative review indicates that Sanda training in youth is associated with improvements in multiple domains of physical fitness (including muscular strength, cardiovascular endurance, flexibility, and coordination), alongside psychological benefits such as mental resilience, focus, and emotional regulation. At the same time, the sport's high-intensity contact nature elevates the risk of acute and overuse injuries, particularly to the lower limbs and upper extremities, which necessitate careful attention to technique, load management, and recovery practices in youth athletes. Across the included studies, there is broad agreement that appropriately structured Sanda programs can enhance health and performance, but effect sizes and reported injury rates vary, likely due to differences in age groups, training volume, competitive level, and methodological quality.

Some investigations emphasize large gains in aerobic capacity and body composition among adolescent Sanda practitioners compared with non-athletes, whereas others report more modest differences or highlight that benefits are contingent on adequate nutritional support and rest. Similarly, research on psychological outcomes generally supports positive effects on self-confidence and stress coping, but these findings are based on small samples and cross-sectional designs, which limit the strength of causal inferences. When interpreted alongside broader evidence from youth combat sports and general youth-sport research, the current literature suggests that Sanda shares many of the physical and psychosocial benefits seen in other martial arts and intermittent, high-intensity sports, while also presenting comparable challenges related to injury prevention, early specialization, and balancing performance goals with long-term development.

## 12 Limitations of the current evidence

Generally, there are several studies on Sanda, but most of them are limited by having cross-sectional designs, small sample sizes from only 1 team or geographical area, and having different testing protocols. Therefore, finding out how Sanda studies can generalize to larger populations or allow for comparisons between studies can be difficult. There have been no longitudinal or interventional studies conducted on the relationship between any specific training or weight management methods used to improve performance or injury outcomes. Women, youth, and those practicing Sanda at the international level have been disproportionately represented in the studies conducted to date, and there have been no studies documenting the training load, recovery methods, or competitive schedules for female Sanda athletes, youth Sanda competitors, and international athletes. To create a better research base regarding performance and health in this sport, it is essential to create standardized testing batteries and develop multi-center collaborations.

## 13 Practical applications

Sanda coaches should develop the training program based on the sport's high-intensity interval characteristics. This means providing a training experience that reflects the competitive format with repeated short-duration striking and wrestling competitions without complete recovery between each competition. The strength and power development program should focus on explosiveness from the lower body for kicking and throwing, speed-strength from the upper body for punching, and trunk rotation power using Olympic lifts, jumping exercises, and the development of powerful medicine-ball throwing techniques when feasible. The incorporation of SAQ and neuromuscular drills aimed at enhancing balance, agility, and quick direction-changing capabilities will enhance the quality of an athlete's offensive and defensive transitions and reduce the risk of athlete injury. The weight-management strategies for Sanda athletes should not promote extreme and rapid weight loss, and instead, should promote a gradual decrease in weight, the use of the services of a registered professional nutritionist, and proper discipline with respect to weight restriction policies to ensure that the athlete is protected from ill health.

## 14 Conclusions and recommendations for future research

Sanda offers huge blessings to youth athletes, promoting both physical and mental improvement. Through its aggregate of striking, kicking, and throwing strategies, Sanda facilitates enhanced cardiovascular fitness, muscular power, coordination, stability, and versatility. The game also contributes to mental resilience, recognition, and emotional regulation, critical characteristics for youth athletes, both outside and inside the sports activities arena. But, like any fight sport, Sanda offers demanding situations, specifically concerning injury risks. Notable injuries, along with strains, sprains, and joint-related troubles, are accepted due to the bodily depth and the excessive nature of education and competition. Additionally, the sport's emphasis on explosive movements and throws can result in overuse injuries, particularly among youth athletes still growing bodily.

To enhance the advantages and reduce the demanding situations related to Sanda for youth athletes, numerous enhancements may be made. First, education applications must be cautiously tailored to every athlete's age, physical maturity, and enjoyment stage. Customized schedules with proper warm-up and funky-down workouts ought to be prioritized to prevent injuries and ensure long-term bodily improvement. Implementing progressive depth is vital to avoid overtraining, even as it still allows youth athletes to increase their vital competencies and patience.

Additionally, greater emphasis should be placed on protection measures, such as the necessary use of protective gear, mainly during sparring and excessive-touch education. More comprehensive injury prevention training for coaches and athletes can help decrease the prevalence of common injuries. Advanced performance monitoring techniques, including biomechanical analysis and physiological tracking, can assist coaches in verifying progress more appropriately, discovering capacity dangers, and altering training loads to prevent burnout and injury. Introducing sports activities science technology to track athlete information, including heart rate, recuperation time, and physical load, will provide real-time insights into education effectiveness and fitness fame.

Destiny studies in Sanda need to raise awareness on improving training protocols, improving safety measures, and refining injury prevention strategies. Exploring the long-time period effect of Sanda on youth athletes' musculoskeletal fitness, cognitive development, and intellectual well-being is likewise essential for expertise how the game influences normal growth. Researchers may want to further check out the function of move-education among Sanda and different martial arts, analyzing how the combination of various techniques impacts performance, harm prevention, and long-term athletic development. Studies on mental conditioning in adolescent Sanda athletes may offer deeper insights into mental instruction, enhancing how youth athletes control pressure, competition strain, and emotional law through high-stakes activities.

In conclusion, while Sanda presents several advantages for youth athletes, specifically in developing properly rounded bodily and mental skills, attention ought to be paid to the game's potential risks. Via progressive education practices, safety protocols, and performance monitoring, Sanda can remain a precious tool in adolescents' athletic improvement, with future studies supporting the refinement and enhancement of its effect on youth athletes.
